# Spontaneous Pneumothorax During Stereotactic Body Radiotherapy for Pulmonary Metastasis From Osteosarcoma: A Case Report and Literature Review

**DOI:** 10.7759/cureus.116388

**Published:** 2026-09-16

**Authors:** Vikash Kumar, Praveen K Bansal, Arun Pandey, Sweta Dhandhi, Shyam Bisht

**Affiliations:** 1 Radiation Oncology, Metro Cancer Institute, Faridabad, IND; 2 Medical Oncology, Metro Cancer Institute, Faridabad, IND; 3 Surgical Oncology, Metro Cancer Institute, Faridabad, IND; 4 Radiation Oncology, Medanta-The Medicity, Gurgaon, IND

**Keywords:** cavitation, deep inspiratory breath hold, osteosarcoma, pneumothorax, pulmonary metastasis, stereotactic body radiotherapy

## Abstract

Pulmonary metastases represent the predominant pattern of distant disease in osteosarcoma and are associated with a spectrum of thoracic complications. Among these, spontaneous pneumothorax is an infrequent but clinically important event. Here, we present the case of a young man who developed spontaneous pneumothorax during the course of stereotactic body radiotherapy (SBRT) for a subpleural pulmonary metastasis delivered using the Deep Inspiratory Breath Hold (DIBH) technique. The pneumothorax was detected on routine image guidance after the fourth fraction of treatment. Despite this unexpected event, the patient remained clinically stable and was successfully managed conservatively with supplemental oxygen, allowing completion of SBRT following a brief treatment interruption. Interval imaging demonstrated rapid cavitary transformation and necrosis of the treated subpleural metastasis, accompanied by complete resolution of the pneumothorax. Although pneumothorax has been described as a rare and typically delayed complication of lung SBRT, its occurrence during the active course of treatment is exceptionally uncommon. Whether the event in our patient resulted from rapid radiation-induced tumor necrosis, the subpleural location of the lesion, DIBH-related pleural stretch, or a combination of these mechanisms remains speculative. This case highlights the importance of vigilance for pneumothorax during SBRT for peripheral pulmonary metastases and illustrates that carefully selected asymptomatic patients may be managed conservatively with successful completion of treatment.

## Introduction

Osteosarcoma is the most common primary malignant tumor of bone [1]. Approximately 20% of patients have metastatic disease at the time of diagnosis, with the lungs representing the predominant site of metastasis in nearly 80%-90% of cases [2]. Despite considerable advances in multimodality treatment, pulmonary metastases remain the leading cause of disease-related morbidity and mortality.

Whenever technically feasible, complete pulmonary metastasectomy remains the preferred local treatment and, when combined with systemic chemotherapy, offers the best chance of prolonged survival [3,4]. However, repeat thoracic surgery is not always possible because of recurrent disease, an unfavorable anatomical location, limited pulmonary reserve, or poor patient performance status. In these situations, stereotactic body radiotherapy (SBRT) has emerged as an effective, non-invasive local ablative option for carefully selected patients with oligometastatic lung disease, achieving excellent local control while preserving normal lung function [5,6].

Patients with pulmonary metastases may develop a range of thoracic complications, including pleural effusion, hemoptysis, airway obstruction, cavitation, and, less commonly, spontaneous pneumothorax [7]. Spontaneous pneumothorax, although uncommon, is a recognized complication of pulmonary metastatic osteosarcoma, particularly in patients with peripheral or subpleural metastatic deposits. Proposed mechanisms include rupture of subpleural metastases, tumor necrosis and cavitation with subsequent pleural disruption, and formation of a bronchopleural fistula [7-10]. Importantly, pneumothorax in this setting may occur as a complication of the metastatic disease itself or following systemic therapy and therefore cannot necessarily be attributed to radiotherapy. Pneumothorax has separately been reported as a rare complication following lung SBRT, with an incidence of approximately 1%-2%; however, reported cases have generally occurred as delayed events after completion of treatment [11,12]. In contrast, the pneumothorax in our patient developed during the active course of SBRT for a subpleural osteosarcoma metastasis. We report this case because of the unusual timing of the event and the serial imaging findings demonstrating subsequent cavitary transformation of the treated lesion. Although the temporal relationship with SBRT is noteworthy, it does not establish causality, and the relative contributions of the underlying subpleural metastasis, tumor response, SBRT, and Deep Inspiratory Breath Hold (DIBH) remain uncertain.

## Case presentation

A 20-year-old male patient with a known diagnosis of high-grade osteogenic sarcoma of the left distal femur initially underwent neoadjuvant chemotherapy followed by limb-salvage surgery with reconstruction. During surveillance, he developed oligometastatic disease involving the left lung, for which he underwent video-assisted thoracoscopic (VATS) wedge resection and metastasectomy. The histopathology confirmed metastatic osteosarcoma.

He was started on chemotherapy; the regimen consisted of gemcitabine 900 mg/m² administered on Days 1 and 8, along with docetaxel 75 mg/m² on Day 8, repeated every 21 days. While on chemotherapy, the follow-up imaging demonstrated recurrent metastasis, measuring approximately 3.4 × 3.5 cm in the left upper lobe abutting the pleura (Figure 1A). The case was discussed in a multidisciplinary tumor board, and in view of the limited pulmonary metastatic burden and previous metastasectomy, the patient was planned for SBRT to the lung lesions.

**Figure 1:**
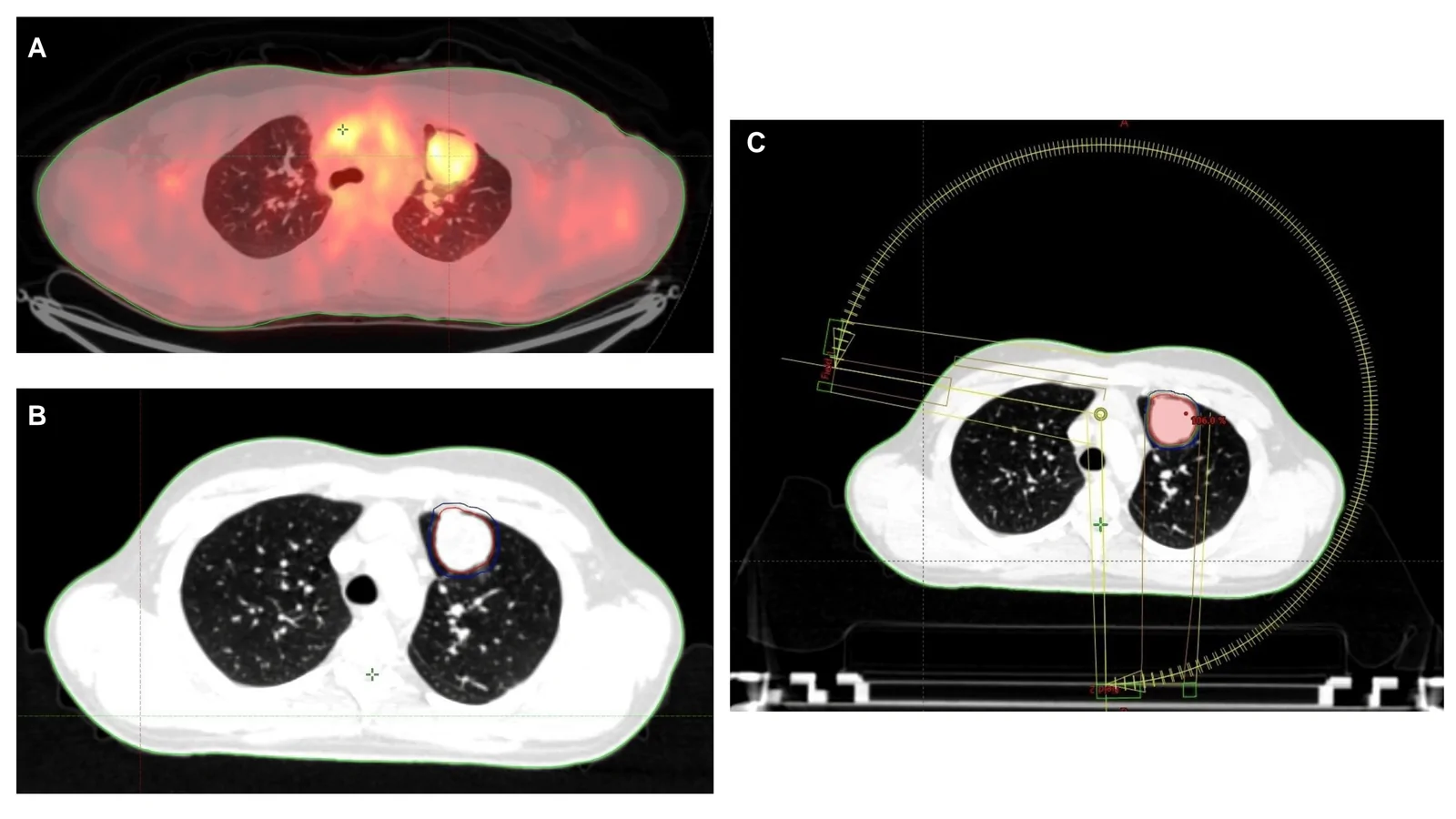
Pretreatment imaging and stereotactic body radiotherapy (SBRT) planning for the left upper lobe pulmonary metastasis. (A) Axial fused PET-CT image demonstrating an intensely FDG-avid, well-circumscribed metastatic lesion in the left upper lobe, located in a peripheral subpleural position with broad-based pleural contact. (B) Axial planning CT image acquired during Deep Inspiratory Breath Hold (DIBH), showing delineation of the gross tumor volume (GTV, red contour) and planning target volume (PTV, blue contour). (C) Axial treatment planning image illustrating highly conformal SBRT dose distribution encompassing the target while maintaining acceptable doses to surrounding normal tissues. FDG: fluorodeoxyglucose; CT: computed tomography

SBRT was delivered using an image-guided volumetric modulated arc therapy (VMAT)-based technique to a dose of 50 Gy in five alternate-day fractions to the planning target volumes (Figures 1B, 1C). The last chemotherapy cycle was completed one week prior to the planned SBRT, and no concurrent chemotherapy was administered during the course of SBRT.

Treatment planning was optimized to achieve adequate target coverage while respecting normal tissue constraints. All relevant dose-volume parameters for the lungs and adjacent organs at risk, including the chest wall and ribs, were within protocol-specified limits and met the recommended constraints as outlined in the American Association of Physicists in Medicine (AAPM) Task Group 101 (TG-101) guidelines [13]. The patient was treated using the DIBH technique to reduce respiratory motion of the target, which enabled accurate target localization and optimal sparing of adjacent normal tissues.

The treatment was initially well tolerated, with satisfactory image guidance and excellent reproducibility on daily cone-beam computed tomography (CBCT) verification (Figures 2A, 2B). However, after the fourth fraction of SBRT, the patient was found to have developed a pneumothorax on routine onboard CBCT imaging with a maximum visceral-to-parietal pleural separation of approximately 4.74 cm on the axial images (Figure 2C).

**Figure 2:**
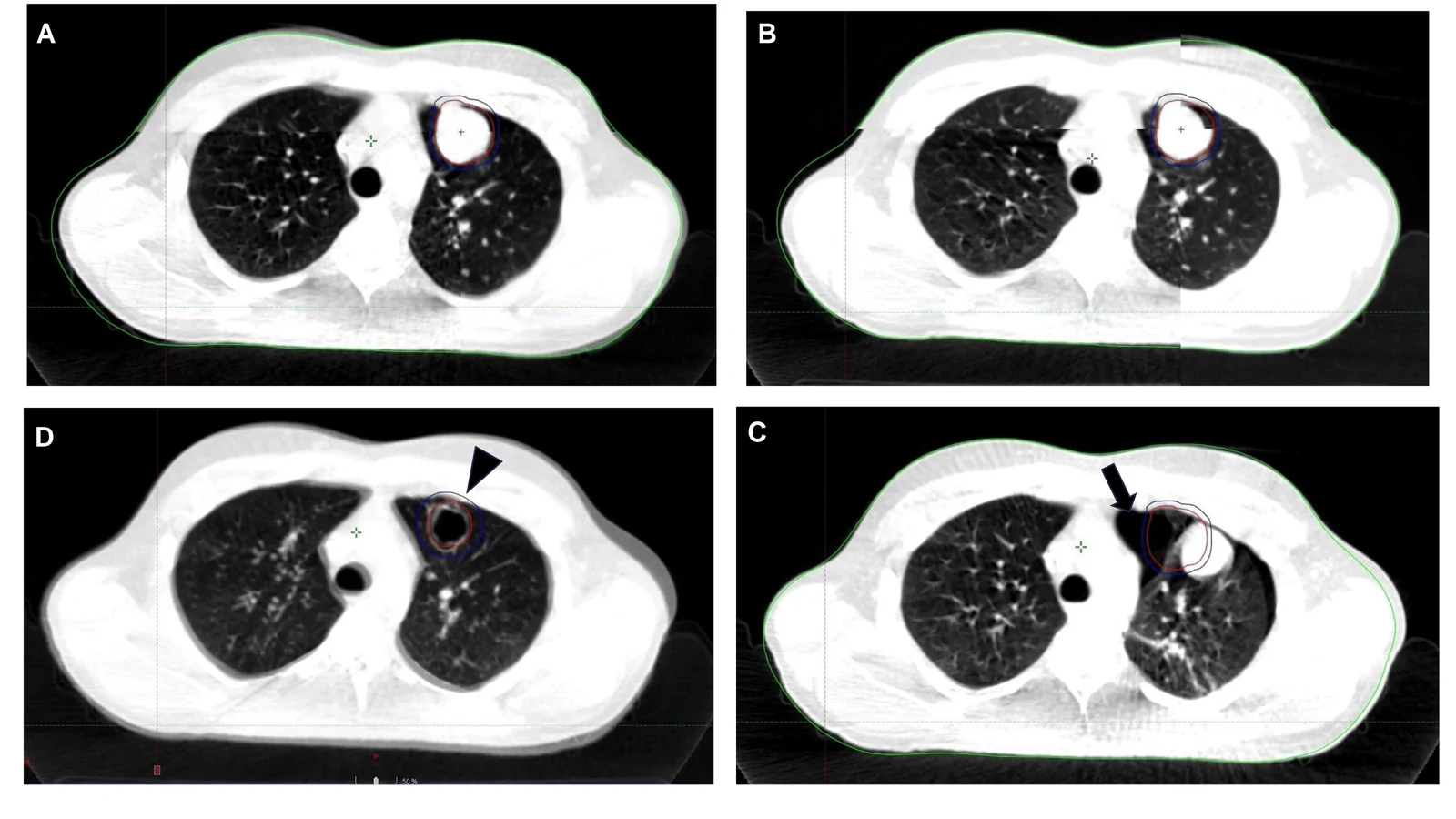
Serial cone-beam computed tomography (CBCT) images demonstrating the evolution of treatment-related pneumothorax during stereotactic body radiotherapy (SBRT). Serial CBCT images acquired during image-guided SBRT demonstrating the development and subsequent resolution of pneumothorax. (A) Pretreatment CBCT before the first SBRT fraction demonstrating satisfactory target localization and image registration. (B) Pretreatment CBCT before the second SBRT fraction showing continued reproducible target positioning with stable image congruence. (C) CBCT acquired before the fifth scheduled fraction demonstrating a newly developed left-sided pneumothorax (arrow) adjacent to the treated subpleural metastasis, with partial separation of the left upper lobe from the anterior chest wall. (D) Repeat CBCT obtained approximately one week later demonstrating marked cavitary transformation and central tumor necrosis (arrowhead), with interval resolution of the pneumothorax, allowing completion of the final SBRT fraction.

Notably, he was asymptomatic except for mild chest pain, without any dyspnea, cough, or oxygen desaturation. Pulmonologist opinion was sought, and he recommended placement of an intercostal chest drain (ICD). However, considering his absence of symptoms and after detailed counseling regarding the risks and benefits, the patient and his family declined invasive intervention.

The patient was therefore managed conservatively with supplemental high-flow oxygen therapy and close clinical and radiological monitoring. Over the ensuing one week, the pneumothorax gradually resolved without any clinical deterioration or requirement for invasive management. Repeat imaging performed for treatment reassessment demonstrated an excellent early response of the irradiated lung metastases, with marked reduction in the solid component and transformation into cavitating necrotic lesions (Figure 2D).

Following conservative management, interval imaging demonstrated resolution of the pneumothorax along with marked cavitary transformation of the treated lesion. As these changes had altered the local thoracic anatomy and potentially the position and geometry of the target, repeat simulation and replanning were performed before treatment was resumed. The target volumes and adjacent organs at risk were re-evaluated on the repeat planning scan, and the fifth and final SBRT fraction was delivered using the revised plan with acceptable dose volume statistics after satisfactory image-guided target verification. The patient subsequently received the fifth and final fraction of SBRT with the same protocol and was discharged with follow-up advice of interval imaging with CT chest planned after six weeks.

## Discussion

While osteosarcoma pulmonary metastases typically present as solid, rounded nodules, often with varying degrees of calcification, a subset may undergo cavitary transformation and develop thick-walled or "bubble-like" appearances on CT [8]. Although spontaneous pneumothorax is an uncommon complication, it has been reported in fewer than 2% of patients with pulmonary metastatic osteosarcoma and is usually associated with peripheral or subpleural metastatic deposits [7,8].

Several mechanisms have been proposed to explain spontaneous pneumothorax in this setting. Rapid tumor necrosis following cytotoxic therapy may result in cavitation and subsequent pleural disruption, particularly in subpleural metastatic lesions [5]. Likewise, treatment-induced cavitation may lead to the formation of a bronchopleural fistula, creating a communication between the bronchial tree and pleural cavity that permits leakage of air into the pleural space [14].

In contrast, pneumothorax following lung SBRT is an uncommon treatment-related toxicity, with a reported incidence of approximately 1%-2%, and most published cases have occurred months to years after completion of radiotherapy rather than during the treatment course [11]. Proposed mechanisms include radiation-induced pleural and parenchymal injury, progressive subpleural fibrosis with rupture of blebs, chest wall or rib injury, and underlying emphysematous lung disease, particularly when treating peripheral lesions abutting the pleura [12]. A comparative summary of the published literature describing spontaneous pneumothorax associated with pulmonary metastases and SBRT is presented in Table 1, highlighting the distinctive clinical features and novelty of the present case.

**Table 1:** Published literature on spontaneous pneumothorax associated with pulmonary metastases and stereotactic body radiotherapy (SBRT). DIBH: Deep Inspiratory Breath Hold

Sl. No.	Author (year)	Study type	Primary disease	Pneumothorax setting	Proposed mechanism	Management/outcome	Relevance to present case
1	Steinhäuslin and Cuttat (1985) [10]	Case series	Various pulmonary metastases	Spontaneous pneumothorax	Bronchopleural fistula secondary to tumor necrosis and cavitation	Variable	First description of pneumothorax related to metastatic pulmonary disease.
2	Le Garff et al. (2001) [7]	Case report	Osteosarcoma	Recurrent spontaneous pneumothorax associated with pulmonary metastasis	Excavated/subpleural pulmonary metastasis with presumed pleural disruption	Surgical excision	Demonstrates the association between subpleural/cavitating osteosarcoma pulmonary metastases and spontaneous pneumothorax.
3	Nakada et al. (2014) [9]	Case series	Osteosarcoma	Secondary spontaneous pneumothorax	Cavitating pulmonary metastases	Surgical management	Demonstrated that pneumothorax may complicate metastatic osteosarcoma and occasionally requires surgical intervention.
4	Alduraibi et al. (2024) [8]	Pictorial review	Osteosarcoma	Imaging review	Cavitary ("bubble-like") pulmonary metastases	Not applicable	Highlights the spectrum of thoracic imaging findings, including cavitating metastases predisposing to pneumothorax.
5	Asai et al. (2013) [11]	Conference abstract	Lung tumor	After SBRT	Radiation-induced pleural injury (presumed)	Not specified	First report describing pneumothorax after SBRT; onset occurred after treatment rather than during treatment.
6	Kang et al. (2015) [12]	Review	Lung cancer	Late SBRT toxicity	Pleural injury, fibrosis, bleb rupture, chest wall injury	Not applicable	Summarizes incidence (1%-2%) and proposed mechanisms of SBRT-associated pneumothorax.
7	Present case	Case report	Osteosarcoma with pulmonary metastasis	During the fourth fraction of SBRT using DIBH	Rapid tumor necrosis, subpleural lesion, possible transient bronchopleural communication; contributory role of DIBH remains speculative	Conservative management with oxygen; SBRT completed successfully	First documented report of pneumothorax developing during the active course of SBRT for pulmonary metastasis from osteosarcoma with serial imaging documenting cavitation and resolution.

To the best of our knowledge, spontaneous pneumothorax developing during the active course of SBRT for pulmonary metastases from osteosarcoma has not been previously reported. In our patient, the close temporal relationship between high-dose hypofractionated radiotherapy, rapid cavitary transformation of the irradiated lesion, and subsequent pneumothorax suggests a plausible treatment-related association. The metastatic nodule was located immediately beneath the visceral pleura with broad-based pleural contact and minimal intervening aerated lung parenchyma. This anatomical relationship may have increased its susceptibility to pleural disruption following rapid tumor necrosis and cavitation, resulting in a transient pleural-alveolar communication and air leak.

The precise mechanism underlying pneumothorax in our patient remains speculative. We hypothesize that the ablative radiation dose accelerated tumor necrosis within the subpleural metastasis, weakening the adjacent visceral pleura. Although unproven, repetitive pleural stretch during treatment with the DIBH technique may have imposed additional mechanical stress on an already compromised pleural surface. The subsequent cavitary transformation of the lesion may also have facilitated the formation of a transient bronchopleural communication, providing a biologically plausible explanation for the unusually early onset of pneumothorax during SBRT. An additional learning point from this case is that selected asymptomatic patients with a stable pneumothorax may be managed conservatively with supplemental oxygen, close clinical observation, and serial imaging, allowing safe completion of curative-intent treatment when clinically appropriate.

## Conclusions

This case describes an unusual occurrence of spontaneous pneumothorax during the active course of SBRT for a subpleural pulmonary metastasis from osteosarcoma. Although the temporal association raises the possibility that SBRT may have contributed to or precipitated the event, a causal relationship cannot be established. The peripheral subpleural location and broad pleural contact of the metastatic lesion itself represented a plausible substrate for spontaneous pneumothorax, and the marked cavitary transformation documented on subsequent imaging occurred after the pneumothorax had already been detected. Any contribution from DIBH also remains speculative. In this individual case, routine image guidance enabled early detection in a minimally symptomatic patient, and conservative management with close clinical and radiological monitoring was followed by resolution of the pneumothorax and subsequent completion of SBRT. This case highlights the importance of recognizing both disease-related and potential treatment-related mechanisms when pneumothorax occurs during SBRT for pleural-based pulmonary metastases.
